# Deficiency in pulmonary surfactant proteins in mice with fatty acid binding protein 4-*Cre*-mediated knockout of the tuberous sclerosis complex 1 gene

**DOI:** 10.1113/expphysiol.2012.069674

**Published:** 2012-11-09

**Authors:** Xinxin Xiang, Fang Yuan, Jing Zhao, Ziru Li, Xian Wang, Youfei Guan, Chaoshu Tang, Guang Sun, Yin Li, Weizhen Zhang

**Affiliations:** 1 Department of Physiology and Pathophysiology, Peking University Health Science Center; Key Laboratory of Molecular Cardiovascular Science, Ministry of EducationBeijing 100191, China; 2 Division of Medicine, Memorial University of Newfoundland, St John'sNewfoundland, Canada; 3 Department of Surgery, University of MichiganAnn Arbor, MI, USA

## Abstract

**New findings:**

- What is the central question of this study?
  Does tuberous sclerosis complex 1–mammalian target of rapamycin (mTOR) signalling regulate the synthesis of surfactant proteins A and B and, if so, can this contribute to the postnatal death of *Fabp4-Tsc1*cKO mice?
- What is the main finding and its importance?
  Our study reveals a novel mechanism for the regulation of alveolar surfactant proteins. Tuberous sclerosis complex 1–mTOR signalling contributes to the regulation of synthesis of surfactant proteins A and B. Deficiency of tuberous sclerosis complex 1 in alveolar epithelial cells may contribute to the postnatal death of *Fabp4-Tsc1*cKO mice.

Tuberous sclerosis complex 1 (TSC1) forms a heterodimmer with tuberous sclerosis complex 2, to inhibit signalling by the mammalian target of rapamycin (mTOR) complex 1 (mTORC1). The mTORC1 stimulates cell growth by promoting anabolic cellular processes, such as gene transcription and protein translation, in response to growth factors and nutrient signals. Originally designed to test the role of TSC1 in adipocyte function, mice in which the gene for TSC1 was specifically deleted by the fatty acid binding protein 4 (FABP4)-*Cre* (*Fabp4-Tsc1*cKO mice) died prematurely within 48 h after birth. The *Fabp4-Tsc1*cKO mouse revealed a much smaller phenotype relative to the wild-type littermates. Maternal administration of rapamycin, a classical mTOR inhibitor, significantly increased the survival time of *Fabp4-Tsc1*cKO mice for up to 23 days. Both macroscopic and microscopic haemorrhages were observed in the lungs of *Fabp4-Tsc1*cKO mice, while other tissues showed no significant changes. Levels of surfactant proteins A and B demonstrated a significant decrease in the *Fabp4-Tsc1*cKO mice, which was rescued by maternal injection of rapamycin. Co-localization of FABP4 or TSC1 with surfactant protein B was also detected in neonatal pulmonary tissues. Our study suggests that TSC1–mTORC1 may be critical for the synthesis of surfactant proteins A and B.

Tuberous sclerosis complex (TSC) is an autosomal dominant genetic disorder characterized by development of hamartomas, which can arise in multiple organs, including brain, kidney, skin and liver (Borkowska *et al.* 2011). Tuberous sclerosis complex is exclusively associated with mutations in either the TSC1 or TSC2 tumour suppressor gene, which encodes the proteins hamartin and tuberin, respectively. These two proteins form a complex and function as a negative regulator for the mammalian target of rapamycin (mTOR) complex 1 (mTORC1) signalling. Interference with TSC1/2 promotes constitutive activation of mTORC1 (Hay & Sonenberg, 2004). The mTORC1 consists of mTOR, mLST8 (also termed G-protein h-subunit-like protein, GhL, a yeast homologue of LST8), raptor (regulatory-associated protein of mTOR), and PRAS40 (proline-rich Akt substrate 40 kDa), and is rapamycin sensitive (Dunlop & Tee, 2009). The mTORC1 regulates translation initiation by phosphorylation of p70 S6 kinase 1 (S6K1) and eukaryotic initiation factor 4E binding protein 1, and functions primarily in the control of a large array of cellular processes, including cell proliferation, growth, differentiation and survival (Iadevaia *et al.* 2012). Recent studies also reveal that mTORC1 signalling is critical in the sensing of nutrient availability, and in the regulation of food intake and energy balance (Cota *et al.* 2008; Lian *et al.* 2008; Cota, 2009; Polak & Hall, 2009; Catania *et al.* 2011).

Adipose tissue, a fat storage depot and an endocrine organ, controls energy metabolism, appetite and fertility in response to nutrients and insulin. Interestingly, the fat body in *Drosophila* controls full-body growth in response to nutrients (Colombani *et al.* 2003). Downregulation of TOR signalling, most probably dTORC1, specifically in the fat body, leads to a significant reduction in overall body size (Zhang *et al.* 2000). A full-body knockout of any component of mTORC1 is embryonically lethal (Gangloff *et al.* 2004; Murakami *et al.* 2004). Mice with adipose-specific knockout of raptor are lean, resistant to diet-induced obesity, and demonstrate an increase in mitochondrial uncoupling in white adipose tissue (Polak *et al.* 2008). In addition, these mice show better metabolic parameters, including improved glucose tolerance and insulin sensitivity, as well as resistance to diet-induced hypercholesterolaemia. The higher insulin sensitivity is attributable to the leanness and to loss of the S6K1 negative feedback regulation to insulin receptor substrate 1 in adipose tissue. This phenotype is similar to that of mice lacking S6K1 (Um *et al.* 2004), the mTORC1 positive effector, and opposite to that of mice lacking eukaryotic initiation factor 4E binding proteins 1 and 2 (Le Bacquer *et al.* 2007), the mTORC1 negative effectors. Consistent with these findings in the adipose-specific raptor knockout mice, rapamycin treatment causes weight loss in gerbils (Fraenkel *et al.* 2008) and prevents weight gain in rats and humans (Rovira *et al.* 2008). All these studies suggest that mTORC1 signalling in adipose tissue is critically involved in the control of whole-body energy metabolism. While most of these studies focus on the mTORC1 and its downstream effectors, little is known about the role of TSC1/2, the important upstream regulators of mTORC1, in the development and function of adipose tissues.

This study was originally designed to explore the effect of TSC1 on adipose tissues. Given that mice homozygous for *Tsc1* or *Tsc2* targeted mutations die by mid-embryogenesis (Kobayashi *et al.* 1999; Onda *et al.* 2002; Wilson *et al.* 2005), we generated a colony of mice in which the *Tsc1* gene was specifically deleted by the fatty acid binding protein 4 (FABP4)-*Cre*, namely *Fabp4-Tsc1*cKO mice. Surprisingly, the *Fabp4-Tsc1*cKO mice died prematurely within 48 h after birth. Histological examination revealed significant haemorrhage, hyperplasia of the alveolar wall and pulmonary atelectasis. Both mRNA and protein levels of surfactant proteins (SP) A and B demonstrated a significant decrease in the *Fabp4-Tsc1*cKO mice. These observations suggest that deficiency in the synthesis of surfactant proteins A and B may impair the postnatal survival of *Fabp4-Tsc1*cKO mice.

## Methods

### Ethical approval

The animals used in this study were handled in accordance with the *Guide for the Care and Use of Laboratory Animals* published by the US National Institutes of Health (NIH publication no. 85-23, revised 1996), and all the experimental protocols were approved by the Animal Care and Use Committee of Peking University.

### Animals and animal care

*Fabp4-Cre* mice that express the *Cre* recombinase gene under the control of the *Fabp4* gene promoter, as well as *Tsc1^lox/lox^* mice, in which exons 17 and 18 of the *Tsc1* gene are flanked by *loxP* sites by homologous recombination, were purchased from the Jackson Laboratory (Bar Harbor, ME, USA). The *Fabp4-Tsc1*cKO mice were generated by breeding *Tsc1^lox/lox^* mice with *Fabp4-Cre* mice. Control experiments were performed using littermate *Tsc1^lox/lox^* animals. Deletion of the *Tsc1* gene was validated by the absence of its mRNA and the elevation of mTOR activity measured by the increased phosphorylation of S6 and mTOR in brown fat tissues. Mice were housed on a 12 h–12 h light–dark cycle. Normal chow and water were available *ad libitum*.

### Rapamycin treatment

Rapamycin (Santa Cruz Biotechnology, Inc., Santa Cruz, CA, USA) was initially dissolved in 100% DMSO, stored at –20°C, and further diluted in DMSO immediately before use. Pregnant mice were randomly divided into two groups. At the near-term pregnancy, mice were injected intramuscularly with rapamycin (1 mg (kg body weight)^−1^ day^−1^) or vehicle for 3 days before the birth of pups. Administration of rapamycin to mother mice at the same dose continued for 2 days after labour. Neonatal mice were closely observed for life signs to assess the survival of the offspring.

### Cell culture and plasmid transfection

A549 cells (American Type Culture Collection) were cultured in growth medium (high-glucose Dulbecco's modified Eagle's medium; Invitrogen, Grand Island, NY, USA) supplemented with 10% fetal bovine serum, 100 units ml^−1^ penicillin and 100 units ml^−1^ streptomycin. A549 cells were seeded 24 h prior to transfection. The plasmids of mTOR–wild-type (mTOR-WT), mTOR kinase death mutant (mTOR-KD) and green fluorescent protein (GFP)-pcDNA3.0 were transfected using TurboTect™*in vitro* Transfection Reagent (Fermantas, Vilnius, Lithuania) according to the manufacturer's protocol.

### Extraction of RNA and quantitative real-time PCR analysis

Total RNA was isolated using the TRIzol reagent (Invitrogen, Carlsbad, CA, USA). Reverse transcription and quantitative real-time PCR were performed as previously described (Li *et al.* 2008; Xu *et al.* 2009). The PCRs were performed in duplicate, and each experiment was repeated between three and five times. Primers used in this study are shown in Table 1.

**Table 1:** List and sequences of primers used in RT-PCR experiments

Protein	Upstream primer (5′–3′)	Downstream primer (5′–3′)	Gene accession number	Size of product (bp)
Mouse surfactant protein A	AACAATGGGAGTCCTCAG	GCCTTCAATCACACCTAAG	NM_023134	217
Human surfactant protein A	CCTGGTATCCCTGGAGAG	CCATTGCTGGAGAAGACCT	NM_001093770.2	194
Mouse surfactant protein B	GTGCCAAGAGTGTGAGGA	AGCAGAGGGTTTGGAACG	NM_147779	291
Human surfactant protein B	ATGCCAAGAGTGTGAGGA	ATGCCGTTTGAGTCAGTC	NM_000542.3	204
Mouse surfactant protein C	GAAACTCAGAAACGCCTA	ACTCGGAACCAGTATCAT	NM_011359.2	271
Mouse HIF-1α	ATAGCTTCGCAGAATGCTCAGA	CAGTCACCTGGTTGCTGCAA	NM_010431.2	101
Mouse VEGF	AGACGGACACACATGGAGGT	AAAGACTCAATGCATGCCAC	NM_009506.2	97
Mouse/human β-actin	ATCTGGCACCACACCTTC	AGCCAGGTCCAGACGCA	NM_007393 NM_001101.3	196

### Western blot analysis

Tissues extracts were immunoblotted with SP A (Santa Cruz Biotechnology, Inc., Santa Cruz, CA, USA), SP B (CST, Beverly, MA, USA), FABP4 (Abcam, Cambridge, MA, USA), glyceraldehyde 3-phosphate dehydrogenase (GAPDH; CST) and β-actin (CST) as previously described (Li *et al.* 2008; Xu *et al.* 2009). The specific reaction was detected using an IRDye-conjugated second antibody for 1 h incubation and visualized using the Odyssey infrared imaging system (LI-COR Biosciences, Lincoln, NE, USA). The signals were quantified using ImageJ software (NIH, Bethesda, MD, USA).

### Immunostaining

The lungs were quickly removed and prepared for immunostaining as previously described (Xu *et al.* 2009). Slides were individually incubated with TSC1 (1:100 dilution; CST) or phosphorylated mTOR (p-mTOR; Ser 2448) antibody (1:100 dilution; CST) in a humid chamber at 4°C overnight. Secondary antibody staining was performed with a biotin-labelled horse anti-rabbit antibody (1:200 dilution) for 1 h at room temperature, followed by incubation with a streptavidin–biotin horseradish peroxidase complex (Vector Laboratories, Burlingame, CA, USA). Immunoreactivity was detected using diaminobenzidine substrate (peroxide substrate kit, SK-4100; Vector Laboratories) for 2–5 min. Slides were then counterstained with Mayer's Haematoxylin before dehydration and mounting. The expressions of TSC1 and p-mTOR were analysed by comparing the staining intensities between different samples with and without the addition of a primary antibody in the same conditions.

### Double immunostaining

Paraffin-embedded sections were incubated overnight with a mixture of rabbit polyclonal antibodies to FABP4 (1:100 dilution) or TSC1 (1:100 dilution) and mouse monoclonal antibody to SP B (1:50 dilution), then incubated with the mixture of the following secondary antibodies: goat anti-rabbit fluorescein isothiocyanate-conjugated IgG (1:100 dilution) and goat anti-mouse Texas Red-conjugated IgG (1:100 dilution). Controls included substitution of primary antibodies with mouse IgG or rabbit IgG. The FABP4 or TSC1 and SP B positive cells were evaluated using Image Plus software (fCoder Group, Inc., Alexandria, VA, USA) to quantify the fluorescence intensity inside the cells. Cells with fluorescence intensity ≥150% of background were considered positive. A total of 15 tissue sections from five mice were evaluated.

### Statistical analysis

Data are expressed as means ± SEM. Data analysis used GraphPad Prism software (GraphPad Software Inc., La Jolla, CA, USA). One-way ANOVA, Student–Newman–Keuls test (comparisons between multiple groups) or Student's unpaired *t* test (between two groups) was used as appropriate. A value of *P* < 0.05 denotes statistical significance.

## Results

### Postnatal death of *Fabp4-Tsc1*cKO mice and the rescuing effect of rapamycin

**Figure 1:**
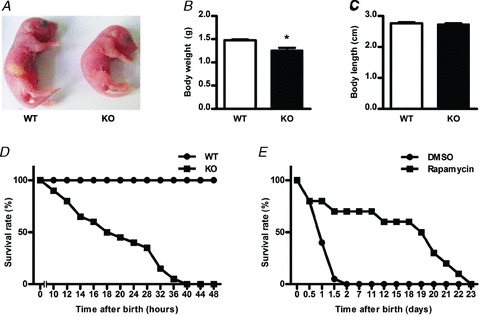
Phenotype of *Fabp4-Tsc1*cKO mice *A*, *Fabp4-Tsc1*cKO mice (KO) are smaller relative to their wild-type littermates (WT). Body weight (*B*) and length from nose to tail base (*C*) were measured and are presented as means + SEM. *D*, life signs were closely observed to assess survival and are presented as survival days and rates. *E* shows the rescuing effect of rapamycin on the survival of *Fabp4-Tsc1*cKO mice. *P < 0.01(*n*= 6–20).

As shown in Fig. 1, *Fabp4-Tsc1*cKO mice were significantly smaller relative to their littermates (Fig. 1*A* and *B*), while the body length showed no difference (Fig. 1*C*). Postnatal death occurred in all *Fabp4-Tsc1*cKO mice. All mice died prematurely within 48 h after birth, with over 60% of death occurring during the first 24 h (Fig. 1*D*). In order to determine the specific effect of *Tsc1* gene deletion in tissues expressing FABP4, we examined the rescuing effect of rapamycin, a specific inhibitor of mTOR signalling, on the survival of *Fabp4-Tsc1*cKO mice. As shown in Fig. 1*E*, administration of rapamycin at a dose of 1 mg kg^−1^ day^−1^ to the near-term pregnant mice significantly increased the survival rate of *Fabp4-Tsc1*cKO mice. The maximal survival time was extended up to 23 days (Fig. 1*E*).

### Expression of FABP4 in neonatal mice and adult mice

**Figure 2:**
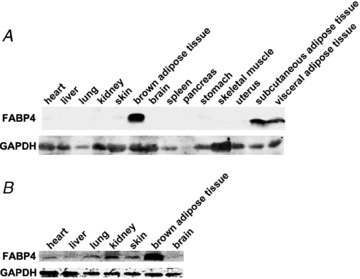
Expression of fatty acid binding protein 4 (FABP4) in neonatal and adult mice Tissues were harvested from neonatal and adult mice, and subjected to SDS-PAGE and Western blot analysis as described in the Methods. Representative results of four individual experiments are shown for the FABP4 expression in a group of tissues derived from adult (*A*) and neonatal mice (*B*). Abbreviation: GAPDH, glyceraldehyde 3-phosphate dehydrogenase.

A series of studies were next performed to determine the potential cause of postnatal death in *Fabp4-Tsc1*cKO mice. We first examined the tissue expression of FABP4. Differential expression of FABP4 was observed in neonatal and adult mice. In adult mice, FABP4 expression was limited to being present only in brown and white adipose tissues (Fig. 2*A*). In contrast, neonatal mice demonstrated a distribution of FABP4 in a wide range of tissues (Fig. 2*B*). In addition to brown and white adipose tissues, FABP4 was detected in lung, heart, skin and kidney, and in liver and brain at a lower level in neonatal mice.

### Pulmonary lesions in *Fabp4-Tsc1*cKO mice

**Figure 3:**
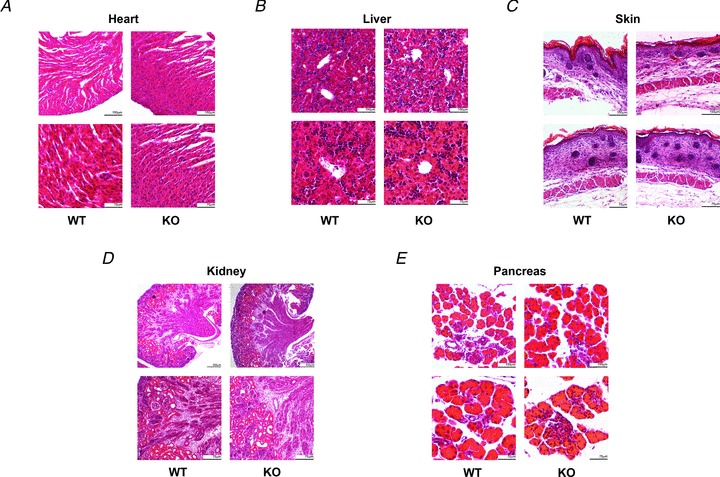
Morphology of tissues of the *Fabp4-Tsc1*cKO mice All tissues were harvested from neonatal mice and processed for Haematoxylin and Eosin staining. Histological evaluation revealed no significant pathological change in heart (*A*), liver (*B*), skin (*C*), kidney (*D*) and pancreas (*E*) in the *Fabp4-Tsc1*cKO mice (KO) compared with their wild-type littermates (WT).

**Figure 4:**
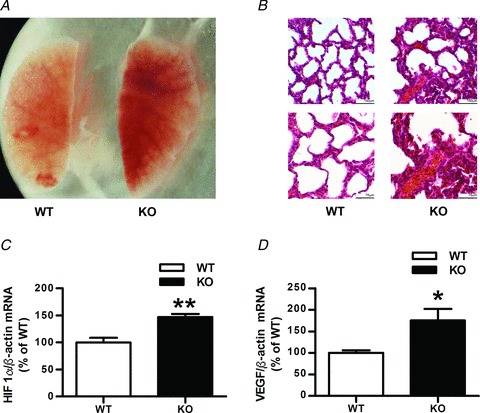
Pulmonary lesions of the *Fabp4-Tsc1*cKO mice The lungs were taken from neonatal *Fabp4-Tsc1*cKO mice (KO) and wild-type littermates (WT). *A*, photographs were taken under the stereoscope. Macroscopic haemorrhage was observed in the lungs of the *Fabp4-Tsc1*cKO mouse. *B*, Haematoxylin and Eosin staining of lung tissue sections shows pulmonary lesions, including haemorrhage, hyperplasia of the alveolar walls and pulmonary atelectasis. The mRNA levels of hypoxia-inducible factor-1α (HIF-1α; *C*) and vascular endothelial growth factor (VEGF; *D*) were markedly increased in the pulmonary tissues in *Fabp4-Tsc1*cKO mice relative to the wild-type animals. *P < 0.05, **P < 0.01 (*n*= 6).

We next examined the histological changes in FABP4-expressing tissues derived from the neonatal *FABP4-Tsc1*cKO mice. No significant histological alteration was observed in tissues such as heart (Fig. 3*A*), liver (Fig. 3*B*), skin (Fig. 3*C*), kidney (Fig. 3*D*) and pancreas (Fig. 3*E*). However, macroscopic haemorrhage was observed in the lungs (Fig. 4*A*). Haematoxylin and Eosin staining confirmed significant pathological lesions in pulmonary tissues, including haemorrhage, hyperplasia of the alveolar walls and pulmonary atelectasis (Fig. 4*B*).

Given that hypoxia-inducible factor-1α (HIF-1α) and vascular endothelial growth factor (VEGF) have been reported to be associated with haemorrhage, we next examined the expression of HIF-1α and VEGF in the lungs of *Fabp4-Tsc1*cKO mice. As shown in Fig. 4*C* and *D*, mRNA levels of HIF-1α and VEGF were markedly increased in the pulmonary tissues in *Fabp4-Tsc1*cKO mice relative to the wild-type animals.

### Expression of TSC1 and p-mTOR in the lung of *Fabp4-Tsc1*cKO mice

**Figure 5:**
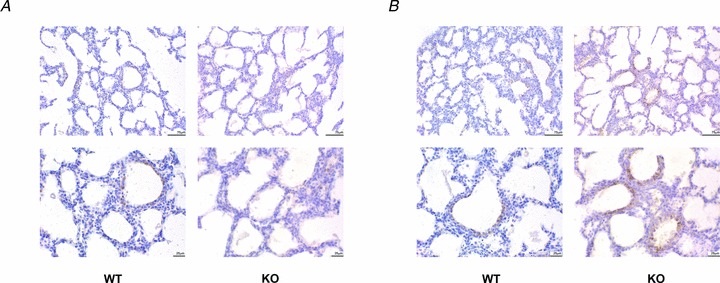
Expression of tuberous sclerosis complex 1 (TSC1) and phosphorylated mammalian target of rapamycin (p-mTOR) in the lungs of *Fabp4-Tsc1*cKO mice The immunoreactivity for TSC1 (*A*) and p-mTOR (*B*) is shown in the lung tissues. A specific signal was observed in the alveolar epithelial cells. Relative to the wild-type mice, TSC1 immunoreactivity was significantly reduced, whereas the p-mTOR signal intensity was higher in the pulmonary tissues of *Fabp4-Tsc1*cKO mice.

In order to validate the deletion of *Tsc1* in the pulmonary tissue of *Fabp4-Tsc1*cKO mice, we next examined the expression of TSC1 and p-mTOR by immunostaining. As shown in Fig. 5*A*, TSC1 immnoreactivity was detected in the pulmonary epithelial cells, whereas no positive signal was demonstrated in the *Fabp4-Tsc1*cKO mice. Consistent with the change in the TSC1 level, *Fabp4-Tsc1*cKO mice demonstrated a much stronger signal for p-mTOR staining in the alveolar epithelial cells (Fig. 5*B*). These results suggest that TSC1 inactivation enhances mTOR activity in the pulmonary epithelia.

### Decrease of alveolar surfactant proteins A and B in *Fabp4-Tsc1*cKO mice

**Figure 6:**
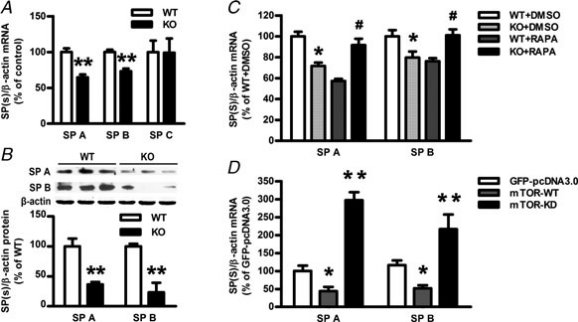
Decrease in the surfactant proteins A and B in the *Fabp4-Tsc1*cKO mice and the rescuing effect of rapamycin The mRNA and protein were extracted from the pulmonary tissues harvested from the *Fabp4-Tsc1*cKO mice (KO) and wild-type littermates (WT; *A–C*) or A549 cells (*D*). Real-time RT-PCR and Western blotting were performed to evaluate the expression of surfactant proteins A, B and C. Results are expressed as means + SEM. *A*, results of surfactant proteins A, B and C mRNA expression in WT and KO. **P < 0.01 (*n*= 6). *B*, representative results of Western blot. β-Actin was used as the loading control. **P < 0.01 (*n*= 6). *C*, the rescuing effect of maternal rapamycin administration on the transcription of surfactant proteins A and B mRNA in WT and KO mice. *P < 0.05 (*n*= 6) compared with WT + DMSO group. #*P* < 0.05 (*n*= 6) compared with KO + DMSO group. *D*, effects of overexpression of mTOR-WT and mTOR-KD plasmids on the mRNA expression of surfactant proteins A and B. Cells trasfected with GFP-pCDNA3.0 were used as the control. *P < 0.05, **P < 0.01 (*n*= 3).

Given that surfactant proteins secreted by type II alveolar epithelial cells play a central role in pulmonary ventilation, levels of alveolar SPs were then examined. As shown in Fig. 6*A*, mRNA levels of pulmonary SP A and SP B were markedly decreased in the pulmonary tissues derived from the neonatal *Fabp4-Tsc1*cKO mice, whereas SP C demonstrated no significant change. This change was associated with a significant reduction in the protein levels of both SP A and SP B (Fig. 6*B*).

Administration of rapamycin at a dose of 1 mg kg^−1^ day^−1^ into the near-term pregnant mice prevented the reduction of SP A and SP B mRNA (Fig. 6*C*).

To further test the effect of mTOR signalling in the regulation of SP A and SP B transcription, A549 cells, a human alveolar basal epithelial cell line, were transfected with mTOR-WT or mTOR-KD plasmids to increase and decrease the mTOR signalling in these cells, respectively. As a control, GFP-pcDNA3.0 was used. As shown in Fig. 6*D*, mRNA levels of SP A and SP B were markedly decreased after activation of mTOR by mTOR-WT plasmid, whereas inhibition of mTOR by mTOR-KD plasmid increased the mRNA levels of SP A and SP B.

### Co-localization of FABP4 or TSC1 with surfactant protein B in alveolar epithelium

**Figure 7:**
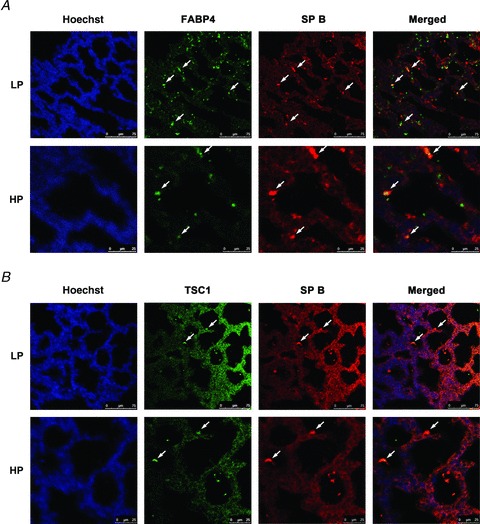
Co-localization of FABP4 or TSC1 with surfactant protein B Images depict FABP4 or TSC1 (green) and surfactant protein B (red) in alveolar epithelial cells. Merged images illustrate co-localization of surfactant protein B and FABP4 (orange; *A*) or TSC1 (*B*). Controls included substitution of primary antibodies with mouse IgG and rabbit IgG. Cells expressing FABP4 or TSC1 and surfactant protein B are indicated by white arrows. Top panels, low magnification (LP); bottom panels, high magnification (HP).

Double immunofluorescent staining was performed to co-localize the expression of FABP4 or TSC1 with SP B in the neonatal lung tissues. As shown in Fig. 7*A*, antibody recognizing FABP4 showed a positive reaction in the alveolar epithelial cells that were also reactive for SP B. Likewise, co-localization of TSC1 immunoreactivity with SP B was observed in the alveolar epithelial cells (Fig. 7*B*). Control antibodies produced no positive signal.

## Discussion

Our present study demonstrates that conditional deletion of *Tsc1* driven by the *Fabp4* promoter causes a deficiency in the synthesis of surfactant proteins A and B, which may at least partly contribute to the postnatal death of *Fabp4-Tsc1*cKO mice. This conclusion is supported by the following observations: (i) *Fabp4-Tsc1*cKO mice die within 48 h after birth; (ii) inhibition of mTORC1 signalling by rapamycin rescues the mice from postnatal death; (iii) a much wider expression of FABP4 is found in neonatal tissues, including the lungs; (iv) levels of alveolar surfactant proteins A and B are significantly decreased in *Fabp4-Tsc1*cKO mice; (v) there exists a negative relationship between mTOR signalling and SP A and SP B transcription in the cultured alveolar epithelial cells; and (vi) FABP4 or TSC1 co-localizes with surfactant protein B in alveolar epithelial cells of the neonatal lungs.

Fatty acid binding protein 4, also known as adipocyte-FABP, was first detected in mature adipocytes and adipose tissue (Reese-Wagoner *et al.* 1999). This protein was originally termed adipocyte P2 (aP2) because of its high sequence similarity (67%) to peripheral myelin protein 2 (M-FABP/FABP8). Originally identified as an adipocyte-specific protein (Spiegelman & Green, 1980), the promoter of *Fabp4* has been widely used to target adipose tissue-specific gene expression in mice (Ross *et al.* 1990; Barlow *et al.* 1997; He *et al.* 2003). Two different lines of *Fabp4-Cre* mice have been used in most published studies. One was developed in Dr Barbara Kahn's laboratory at Harvard University (Abel *et al.* 2001) and the other in Dr Ronald Evans's laboratory at the Salk Institute (He *et al.* 2003). In both lines, *Cre* activity is expressed in white and brown adipose tissues in adult mice. Our study used the latter line, which is commercially available from Jackson Laboratory (stock no. 005069).

Most *Fabp4-Cre*-mediated gene knockout mice can survive, but there also are some exceptions. Ablation of the *Vhl* gene in adipose driven by the *Fabp4* gene promoter exhibits embryonic lethality at embyonic day 14.5–18.5, which results from haemorrhages in the brain and liver induced by the upregulation of HIF-1α/VEGF activity (Zhang *et al.* 2012). In addition, Mudhasani *et al.* (2011) showed that mice with *Fabp4-Cre*-driven deletion of *dicer* gene typically died within 3 weeks after birth, shortly before weaning. These results suggest an expression of FABP4 outside the adipose tissues. Indeed, recent studies have demonstrated a wider expression of FABP4 in an array of cells and tissues, including macrophages upon their differentiation from monocytes, human bronchial epithelial cells, arterial endothelial cells, trophoblasts, liver, skeletal muscle fibres, lipoblastoma and liposarcoma, and human urothelial carcinomas (Shum *et al.* 2006; Biron-Shental *et al.* 2007; Lee *et al.* 2007; Ferrell *et al.* 2008; Boiteux *et al.* 2009; Elmasri *et al.* 2009; Scifres *et al.* 2011). Consistent with these findings, our study suggests that FABP4 is present in a wide range of tissues during development and becomes adipocyte specific in adulthood. Taken together, all these findings may challenge the previous concept on the use of *Fabp4* promoter to target adipose tissue-specific gene expression during development.

In our animal model of *Fabp4-Tsc1*cKO mice, specific deletion of the *Tsc1* gene driven by *Fabp4* promoter causes postnatal death. Conditional deletion of *Tsc1* driven by the *Fabp4* promoter is confirmed by the absence of TSC1 mRNA and activation of mTOR as measured by increased phosphorylation of S6 and mTOR in the brown adipose tissues (data not shown). Previous studies have demonstrated that either TSC1 or TSC2 is critical for survival (Tomasoni & Mondino, 2011). Mice homozygous for *Tsc1* targeted mutations die by mid-embryogenesis, while a significant number of heterozygous (*Tsc1*^+/−^) mice on the C57BL/6 background die in the postnatal period, normally at 1–2 days, from unknown causes (Wilson *et al.* 2005). In humans, mutation of either the *Tsc1* or the *Tsc2* gene causes tuberous sclerosis complex, a multisystem genetic disease, which is commonly characterized by neurological symptoms, such as seizures, autism, mental retardation and learning disabilities. While neuronal dysfunction is common in tuberous sclerosis complex, it is unlikely that the dysfunction of the central nervous system is the leading cause of embryonic or neonatal death. In a mouse tuberous sclerosis epilepsy model, conditional deletion of *Tsc1* in astrocytes induces epilepsy commonly observed in tuberous sclerosis complex patients, and these mice survive until adulthood (Uhlmann *et al.* 2002). The present study reveals a novel mechanism for the postnatal death of *Fabp4-Tsc1*cKO mice. Significant pathological changes, which include haemorrhage, hyperplasia of the alveolar walls and pulmonary atelectasis, are observed in these mice. The pulmonary haemorrhage may be due to the defect in the blood vessel structure induced by HIF-1α activation. A high level of HIF-1α has been reported to stimulate the expression of VEGF, which subsequently increases capillary permeability (Weis & Cheresh, 2005; Zhang *et al.* 2012).

Neonatal respiratory distress syndrome is caused by inadequate amounts of pulmonary surfactant due to delayed lung development (Avery & Mead, 1959). Surfactant, a lipoprotein comprised of phospholipids (∼80%), cholesterol (∼10%) and proteins (∼10%), functions to reduce surface tension and to prevent alveolar collapse at end expiration (Weaver & Whitsett, 1991). Surfactant deficiency or dysfunction is associated with the occurrence and development of many pulmonary diseases, such as neonatal respiratory distress syndrome, acute respiratory distress syndrome, asthma and chronic obstructive pulmonary disease (Wright, 2003). Four types of surfactant proteins (A, B, C and D) have been reported (Weaver & Whitsett, 1991). Surfactant protein A is the most abundant, while SP B is crucial for the adsorption of the surfactant film at the alveolar air–liquid interface (Whitsett & Weaver, 2002). Surfactant protein B has been demonstrated to be critical for normal gas exchange in the perinatal period and is therefore indispensable for neonatal survival. Our study suggests that the neonatal death of *Fabp4-Tsc1*cKO mice may result from a deficiency in the synthesis of SP A and SP B in *Fabp4-Tsc1*cKO mice. First, both the mRNA and the protein levels of SP B are markedly reduced in the *Fabp4-Tsc1*cKO mice. Maternal administration of rapamycin prevents the reduction of SP B induced by the activation of mTOR signalling and significantly extends the survival time of the *Fabp4-Tsc1*cKO mice. Second, an alteration in mTOR signalling directly modulates the transcription of SP B in cultured alveolar epithelial cells. Third, FABP4 or TSC1 co-localizes with SP B in the alveolar epithelial cells. It is well known that type II cells synthesize SP A and SP B in the lung. Our studies indicate that TSC1–mTOR signalling may simultaneously modulate the synthesis of both SP A and SP B in these cells. Taken together, all these observations suggest that TSC1 may be involved in the regulation of SP A and SP B, and specific deletion of *Tsc1* driven by the *Fabp4* promoter may impair the synthesis of SP A and SP B in the alveolar epithelial cells. This finding is consistent with the previous reports by Ikeda *et al.* (2011), in which aberrant activation of the Akt–mTOR signalling pathway in lung epithelium causes infant respiratory distress syndrome, and by Miakotina *et al.* (2002), in which the rapamycin-sensitive phosphoinositide 3-kinase–S6K1 signalling pathway is demonstrated to mediate insulin-induced inhibition of SP A mRNA levels in lung epithelial cells.

In conclusion, our studies suggest a novel mechanism for the regulation of alveolar surfactant proteins. Tuberous sclerosis complex 1 contributes to the regulation of synthesis of surfactant proteins A and B. Deficiency of TSC1 in alveolar epithelial cells may partly contribute to the postnatal death of *Fabp4-Tsc1*cKO mice.
